# Early hypotonia and visual regression as presenting features of peroxisome biogenesis disorder: an Egyptian case report

**DOI:** 10.1186/s12887-026-06931-2

**Published:** 2026-05-18

**Authors:** Abdelrahim A. Sadek, Mohammed A. Aladawy, Khaled Hassan, Tarek M. M. Mansour, Mohamed Naser, Mohamed M. Elmoursy, Sohieb Hedewy, Ahmed Hagag, Kawashty Ragab Mohamed, Elsayed Abdelkreem

**Affiliations:** 1 Neuropsychiatry Unit, Department of Pediatrics, Faculty of Medicine, Sohag University, Sohag, Egypt; 2 Neurology Unit, Department of Pediatrics, Faculty of Medicine, Al-Azhar University, Assiut, Egypt; 3 Department of Radio-diagnosis, Faculty of Medicine, Al-Azhar University, Assiut, Egypt; 4 Audio-vestibular Medicine Unit, Department of ENT, Faculty of Medicine, Al-Azhar University, Assiut, Egypt; 5 Faculty of Medicine, Al-Azhar Assiut University, Assiut, Egypt; 6 Department of Neurology, Faculty of Medicine, Al-Azhar University, Assiut, Egypt; 7 Department of Pediatrics, Faculty of Medicine, Sohag University, Sohag, Egypt

**Keywords:** Peroxisome biogenesis disorder, *PEX12* gene, Zellweger spectrum disorder, Developmental regression, Optic atrophy, Consanguinity, Whole-exome sequencing, Variant, Egypt

## Abstract

**Introduction:**

Peroxisome biogenesis disorders (PBDs) are rare autosomal recessive neurodegenerative diseases caused by variants in Peroxin (*PEX)* genes, leading to defective peroxisome assembly and multisystem dysfunction. The *PEX12* variant NM_000286.3: c.1047_1049del (NP_000277.1: p.Gln349del) has been described almost exclusively in Egyptian patients, suggesting a founder effect. We present the first Egyptian case with this variant showing bilateral optic atrophy, expanding the known clinical phenotype of *PEX12*-related PBD.

**Case presentation:**

A six-year-old Egyptian boy, born to first-cousin parents, presented with early hypotonia, developmental regression, visual inattention, and sensorineural hearing loss. Early milestones were initially normal, followed by progressive motor and cognitive decline beginning at age three. Ophthalmologic examination revealed bilateral optic atrophy, and audiologic testing confirmed profound sensorineural hearing loss. Brain Magnetic Resonance Imaging (MRI) showed bilateral periventricular and cerebellar white matter hyperintensities, with restricted diffusion in the involved areas and corpus callosum involvement. Laboratory studies were unremarkable, but trio whole-exome sequencing identified a homozygous *PEX12* deletion (c.1047_1049del), confirming a diagnosis within the Zellweger spectrum of peroxisome biogenesis disorders. The patient was managed with supportive multidisciplinary care, including levetiracetam for seizure control, physiotherapy, nutritional support, and regular follow-up. At one-year follow-up, seizures were controlled, but severe neurological disability persisted.

**Conclusion:**

This case highlights a rare *PEX12*-related peroxisome biogenesis disorder with optic atrophy—an unreported feature of this variant—broadening its phenotypic spectrum. It underscores the diagnostic value of whole-exome sequencing when biochemical tests are inconclusive and emphasizes the need for increased awareness of founder variants in populations with high consanguinity to enable earlier diagnosis, counseling, and targeted screening.

## Background

Peroxisome biogenesis disorders (PBDs) are a heterogeneous group of autosomal recessive metabolic diseases characterized by impaired peroxisome (*PEX*) formation and dysfunction of peroxisomal enzymes, leading to multisystem involvement. The prevalence of Peroxisome Biogenesis Disorders-Zellweger Spectrum Disorders **(**PBD-ZSD) is estimated to be 1 in 75,000 live births worldwide, mostly in the infancy and neonatal period [1].

The disease spectrum is classified into two groups. (A) Zellweger spectrum disorder (ZSD), which is subdivided into: (1) Zellweger syndrome (severe form characterized by neuronal migration defects in the brain); (2) neonatal adrenoleukodystrophy (intermediate form characterized by damage to the myelin sheath, surrounding nerve cells in the brain); and (3) infantile Refsum disease (milder form characterized by damage to the white matter of the brain and affect motor movements) [2]; and (B) Rhizomelic chondrodysplasia punctata (impairs normal development of long bones in the body) [3]. The etiopathogenesis of PBD–ZSD syndrome is an autosomal recessive disorder caused by a variant of the *PEX* genes. *PEX* genes comprise a group of 16 genes, which are: *PEX1*,* PEX2*,* PEX3*,* PEX5*,* PEX6*,* PEX7*,* PEX10*,* PEX11A*,* PEX11B*,* PEX11G*,* PEX12*,* PEX13*,* PEX14*,* PEX16*,* PEX19*,* and PEX26*, responsible for producing peroxin. These genes have a principal role in the proper development of peroxisome assembly [4]. Approximately 70% of PBD-ZSD cases arise as a result of a variant in the *PEX*1 gene located on chromosome 7q21.2 [5]. Disruption of peroxisomal metabolism leads to the accumulation of very-long-chain fatty acids (VLCFA), phytanic acid, and bile acid intermediates, resulting in neurodegeneration, hepatic dysfunction, and other systemic manifestations [5, 6].

Despite advances in molecular diagnostics, genotype–phenotype correlations in *PEX12*-related ZSD remain poorly defined due to the rarity of this variant. Herein, we describe an Egyptian case of PBD caused by a homozygous *PEX12* variant – NM_000286.3: c.1047_1049del (NP_000277.1: p.Gln349del) – presenting with early hypotonia, developmental regression, and visual impairment, thereby expanding the phenotypic and geographic spectrum of this rare disorder.

## Case presentation

### Patient information

**Fig. 1:**
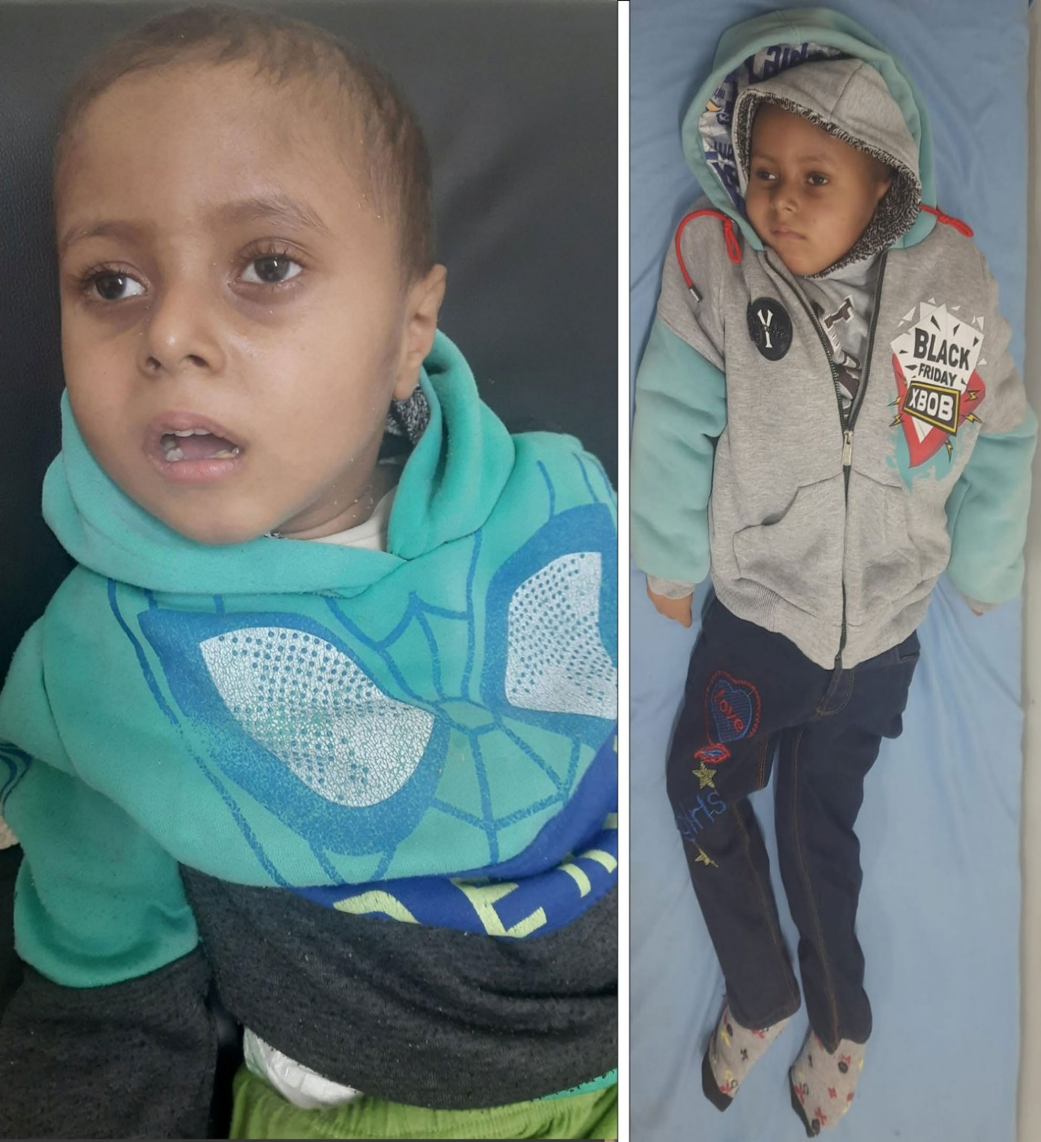
Clinical photograph of the patient showing hypotonic posture, poor head control, and visual inattention consistent with regression of motor and visual functions

A six-year-old Egyptian boy was referred to our pediatric neurology service for progressive neurodevelopmental regression and new-onset seizures. The patient was the youngest of three siblings born to first-cousin parents of Arab descent (Fig. 1). There was no family history of similar neurologic or developmental disorders.

### Birth and perinatal history

The child was delivered at term by cesarean section after an uncomplicated pregnancy; birth weight was 2.8 kg, and Apgar scores were 5, 8, and 9 at 1, 5, and 10 min, respectively. He had an unremarkable neonatal course without the need for neonatal intensive care or documented neonatal jaundice.

### Developmental history

During the first year of life, the patient attained expected early milestones: social smile at 2 months, laughter at 4 months, recognition of his mother at 6 months, independent sitting at 8 months, walking with support at 11 months, and his first spoken syllable at 13 months. Growth parameters recorded at 6 months were weight 6.5 kg, length 62 cm, and head circumference 41.5 cm. Developmental milestones were appropriate for the child’s age, including rolling over, sitting with support, a mature pincer grasp, the use of 2–3 spoken words, and waving “bye-bye.” There was no history of neonatal jaundice, hepatomegaly, or hospital admissions during the first year of life.

The mother noted delayed independent walking, which was not achieved until 2 years of age. At approximately 3 years of age, the child began to lose previously acquired motor abilities, became hypotonic, and was unable to stand independently. With serial follow-up, the patient became visually inattentive.

Regression progressed rapidly: within 3 months, he could no longer sit unsupported, and by 4 years of age, he had lost head control and all meaningful speech, communicating only through non-specific vocalizations. This progressive deterioration raised suspicion of an underlying metabolic, peroxisomal, lysosomal, or mitochondrial disorder, prompting further neuroimaging and metabolic investigations.

### Ophthalmologic and auditory findings

**Fig. 2:**
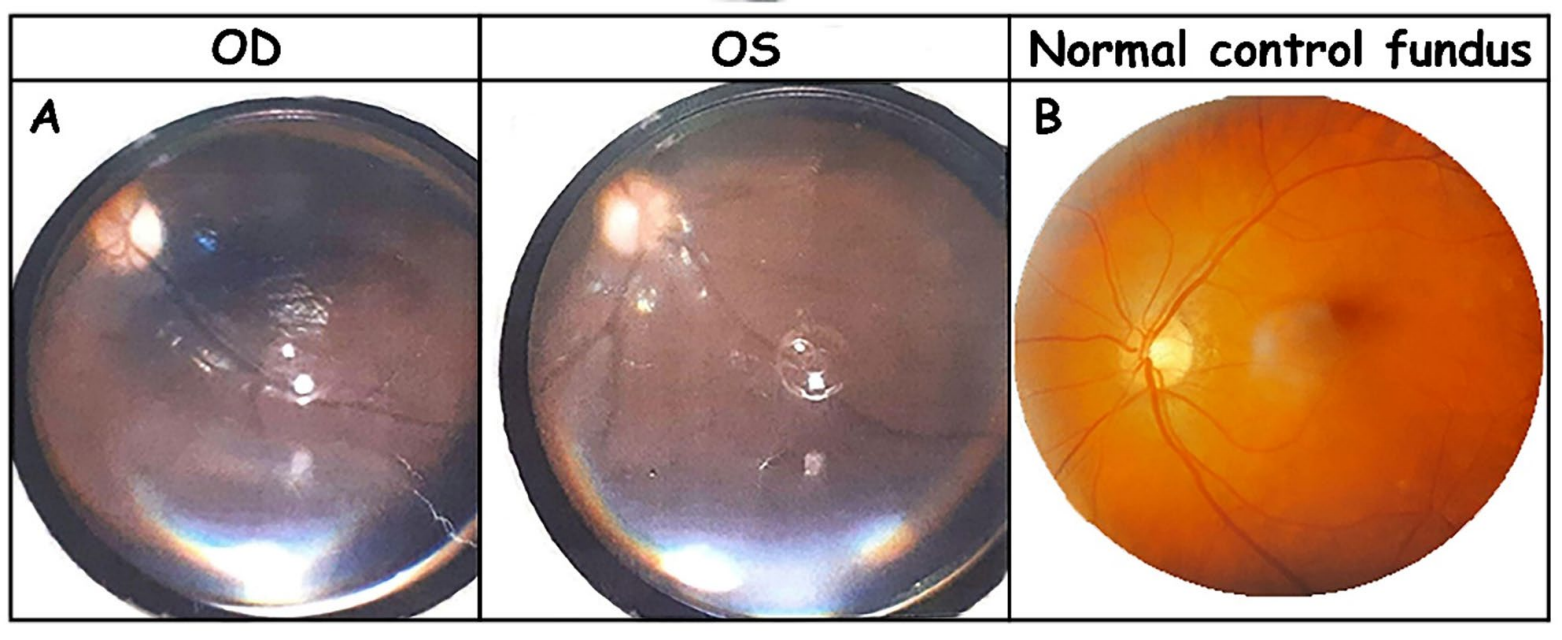
Fundus photographs comparing optic atrophy and a normal control. (**A**) Fundus photographs of the right (OD) and left (OS) eyes demonstrating bilateral optic atrophy, characterized by optic disc pallor, blurred margins, and loss of the normal neuroretinal rim. (**B**) Fundus photograph of a normal control eye showing a healthy optic disc with normal coloration, sharp margins, and preserved neuroretinal rim

Ophthalmologic assessment at the age of 4 revealed bilateral optic atrophy, and the patient was visually inattentive on bedside testing (Fig. 2). Sensorineural hearing loss was documented by objective audiologic testing.

### Neurological and systemic manifestations

Recently, the patient developed recurrent, afebrile generalized tonic-clonic seizures lasting about 10 min, occurring up to seven times per month. Levetiracetam was initiated with subsequent seizure control.

Following a seizure episode, the family reported swelling of the left thigh; radiography showed a mid-shaft femoral fracture that healed with casting over 30 days (orthopedic records). Feeding was normal in early childhood, but progressive dysphagia and drooling developed after 4 years old. There was no history of aspiration pneumonia or prolonged hospital admissions.

### Clinical examination

At 6 years of age, the child was bedridden and non-communicative with absent eye contact. Anthropometric measures were below the 3rd percentile: weight 14 kg, length 92 cm, and head circumference 48 cm. Neurological exam showed generalized hypotonia that is more prominent in the lower than the upper limbs in association with exaggerated deep tendon reflexes and positive Babinski sign.

### Neuroimaging findings

**Fig. 3:**
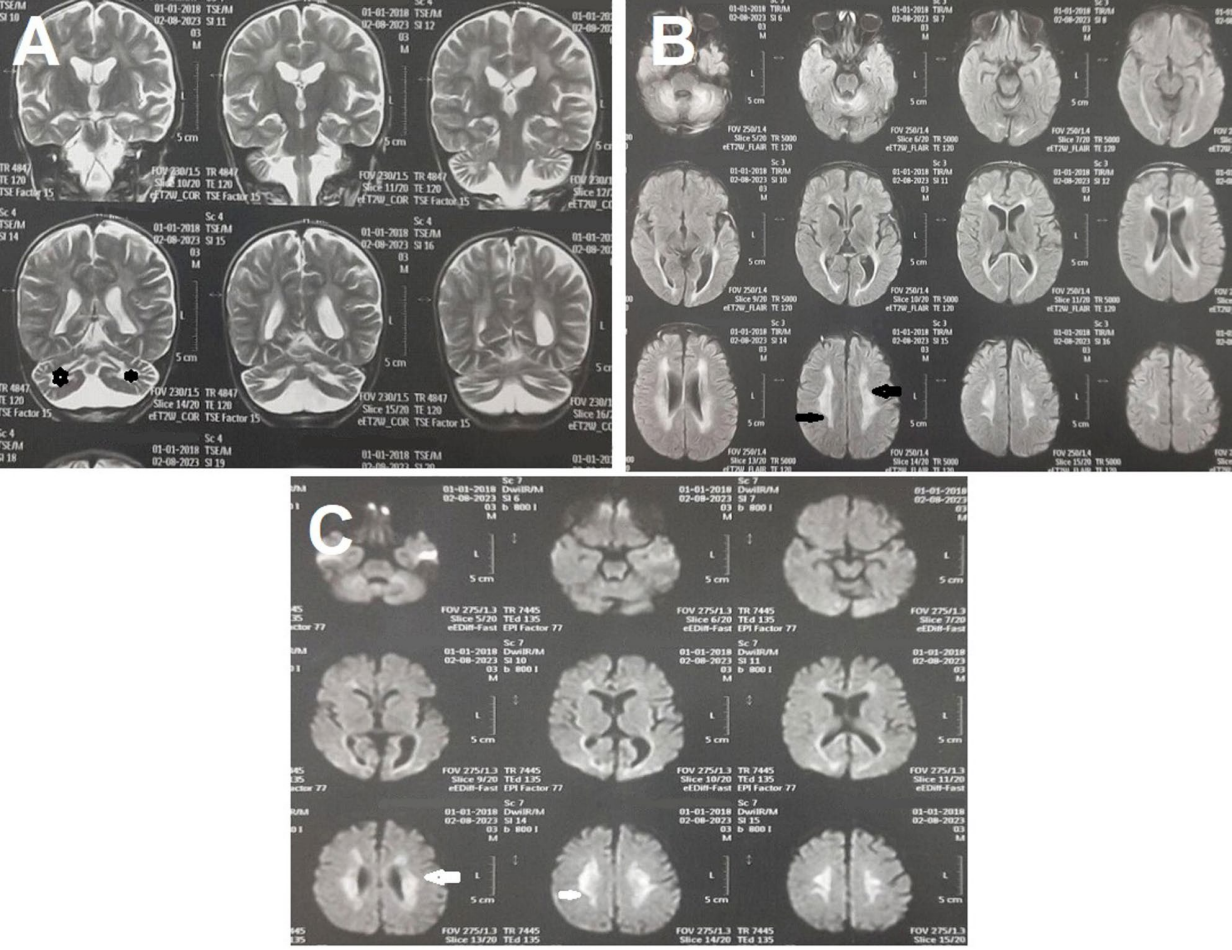
Coronal T2 (**A**), axial FLAIR (**B**), and DWI (**C**) of the brain show bilateral peri-ventricular areas of increased signal (black arrow) and corpus callosum hyperintense signal involving the splenium and genu as well as increased signal of the central cerebellar area, including the dentate nucleus (asterisks). On DWI, there is restricted diffusion in the involved areas (white arrow)

Brain magnetic resonance imaging (MRI) performed at 3 years of age showed bilateral periventricular areas of increased T2/FLAIR signal (black arrow) and hyperintense signal in the corpus callosum involving the genu and splenium, as well as increased T2/FLAIR signal in the central cerebellar region, including the dentate nuclei (asterisks). On diffusion-weighted imaging (DWI), there was restricted diffusion in the involved areas (white arrow). These findings are illustrated in Fig. 3.

### Neurophysiological studies

Electroencephalography revealed generalized background slowing. Auditory brainstem response testing showed severe-to-profound bilateral sensorineural hearing loss at least at the frequency region 2–4 kHz NB, with bilateral failed response in Transient Evoked Otoacoustic Emissions. EMG and nerve conduction studies were within normal limits.

### Laboratory investigations

Laboratory investigations demonstrated AST 18 U/L, ALT 22 U/L, total bilirubin 0.9 mg/dL, direct bilirubin 0.1 mg/dL, creatinine 0.8 mg/dL, ammonia 38 µmol/L, and lactate 19. Lipid profile, acylcarnitine panel by tandem mass spectrometry, and urinary organic acid analysis by gas chromatography–mass spectrometry were unremarkable.

### Genetic analysis

Given the clinical picture of progressive hypotonia, sensorineural hearing loss, optic atrophy, and characteristic MRI abnormalities, genomic testing was pursued. Trio whole-exome sequencing identified a homozygous in-frame deletion in the *PEX12* gene (c.1047_1049del) confirming a diagnosis within the Zellweger spectrum of peroxisome biogenesis disorders caused by PEX gene defects. The parents were presumed heterozygous carriers based on the autosomal recessive inheritance pattern and the presence of a homozygous variant in the proband born to consanguineous parents. Variants in *PEX12* are established causes of peroxisome biogenesis disorders and are consistent with the patient’s phenotype.

According to the American College of Medical Genetics and Genomics (ACMG) variant interpretation guidelines, this variant satisfies the criteria PM2 (absence or extremely low frequency in population databases), PM3 (detected in trans with a pathogenic variant in recessive disorders), PM4 (protein length change due to in-frame deletion), and PP5 (reputable source reports variant as pathogenic), supporting classification as a likely pathogenic variant.

Based on the molecular findings and the relatively delayed onset of neurological regression compared with classic neonatal Zellweger syndrome, the clinical presentation in this patient is most consistent with Peroxisome biogenesis disorder type 3B (PBD3B), representing a delayed-onset phenotype.

### Management and follow-up

Management has been supportive and multidisciplinary. The patient is followed by pediatric neurology, ophthalmology, audiology, nutrition, and physiotherapy teams; interventions have included levetiracetam for seizure control (initiated at 20 mg/kg/day and titrated to a maintenance dose of 50 mg/kg/day), physiotherapy for maintenance of function, nutritional counseling to manage dysphagia, and orthopedic care for fracture management. There is no specific curative therapy for peroxisome biogenesis disorders; care focuses on symptomatic treatment and surveillance for complications.

At 12-month follow-up, the patient remained seizure-controlled on levetiracetam with no reported drug-related adverse effects, but with continued severe motor and sensory impairment and dependence for activities of daily living. Peroxisome biogenesis disorders are progressive conditions with variable survival that often result in significant neurological disability; prognosis depends on the severity and specific molecular defect. The patient’s clinical course, investigations, and outcomes are summarized in Table 1.

**Table 1:** Chronological summary of the patient’s clinical presentation, diagnostic work-up, and management outcomes

Age	Development / Events	Clinical Findings	Diagnostic / Therapeutic Actions	Outcomes
Birth	Full-term C-section, Apgar 5/8/9, BW 2.8 kg	Normal	None	Normal neonatal period
0–12 months	Normal milestones (smile 2 mo, sit 8 mo, walk with support 11 mo, speak “baba” 13 mo)	Normal growth and development	Routine care	Normal
2 years	Could not walk independently	Mild hypotonia	Observation	No intervention
3 years	Lost ability to stand/sit, became hypotonic	Regression of motor milestones	Brain MRI, metabolic work-up	MRI: white matter hyperintensity
4 years	Lost speech and visual contact	Bilateral optic atrophy	Fundus exam, ABR	Bilateral sensorineural hearing loss
5 years	Progressive regression	Severe hypotonia	Supportive physiotherapy	No improvement
6 years	Recurrent afebrile seizures	GTC convulsions, femoral fracture	EEG, X-ray, Levetiracetam 20 mg/kg/day	Seizure control, healed fracture

## Discussion

Our case report describes an Egyptian case of peroxisome biogenesis disorders caused by a homozygous variant in the *PEX12* gene variant (c.1047_1049del), presenting with progressive neurodevelopmental regression, optic atrophy, sensorineural hearing loss, and characteristic neuroimaging abnormalities. This variant is rare outside Egypt, with only 20 cases reported, 19 of which were in Egypt [7, 8], suggesting a possible founder effect or population-specific clustering. ClinVar classified this variant as likely pathogenic (ClinVar ID 556045). The mode of Inheritance is Autosomal recessive (OMIM: 614859), which often leads to homozygous presentation. However, a case reported by Zaki et al. (2020) exhibited a heterozygous variant with a milder clinical presentation.

Limited case series suggest that truncating or otherwise null-effect *PEX12* alleles generally produce classic, early‐onset Zellweger phenotypes, whereas hypomorphic alleles can lead to attenuated disease. Gootjes et al. reported four patients with frame‐shift or early‐stop *PEX12* mutations predicting loss of the zinc‐binding domain who all had severe ZS/NALD, whereas a fifth patient with only missense changes preserving that domain had a milder IRD‐type phenotype [9]. Similarly, a homozygous *PEX12* missense (p.R34S) was found in two unrelated patients who showed only mild infantile cholestasis and hypotonia with mosaic peroxisomal function [10]. By contrast, the founder in‐frame deletion c.1047_1049del, observed predominantly in Egyptian families, has been uniformly caused severe ZSD manifestations, including early regression, hypotonia, cerebellar atrophy, with only a single compound‐heterozygote (bearing another *PEX12* mutation) showing a later‐onset, milder course [8]. These findings align with the general principle described in GeneReviews that *PEX* variants abolishing protein function result in more severe disease, whereas alleles with residual activity produce attenuated phenotypes [9, 11]; In the present case, the onset of developmental regression at approximately three years of age and survival beyond early childhood suggest a delayed clinical phenotype consistent with Peroxisome biogenesis disorder type 3B (PBD3B) rather than the classic early infantile PBD3A form. Nonetheless, formal genotype–phenotype correlations for PEX12 remain incompletely defined.

The patient has a history of consanguineous (cousin) parents, which is known to markedly increase the risk of autosomal recessive disorders, including peroxisome biogenesis disorders [12]. The initial normal milestones, followed by regression, align with the progressive nature of Zellweger spectrum disorders. This may be due to neurodegeneration caused by the accumulation of metabolic derangements [13].

Our patients had progressive hypotonia, gait disturbance, impaired communication skills, seizures, and profound sensorineural hearing loss; these findings are consistent with previous reports by Zaki et al. Additionally, this is aligned with the manifestation of Zellweger spectrum disorders (ZSD), particularly in early-onset cases [8, 14–16]. Brain MRI revealed bilateral periventricular and cerebellar white matter hyperintensities, as well as corpus callosum involvement—findings typical of early-onset ZSD [8, 17, 18].

Optic atrophy observed in our patient appears to represent a novel finding for the homozygous *PEX12* c.1047_1049del variant, although ocular involvement has been frequently reported in other *PEX12*-related ZSD cases. Zellweger spectrum disorders are well known to present with a wide range of ophthalmologic manifestations, including nystagmus, retinopathy, corneal clouding, and cataract formation. In the Egyptian cohort carrying the same founder variant, ocular abnormalities were common, with nystagmus reported in 15 of 20 patients (75%), often becoming more pronounced with age [8]. Visual impairment was documented in nine patients (45%), predominantly due to pigmentary retinopathy in five cases and corneal clouding in two cases, highlighting the vulnerability of retinal structures to peroxisomal dysfunction. These retinal dystrophic changes are consistent with the essential role of peroxisomes in retinal lipid metabolism, where impaired degradation of very-long-chain fatty acids contributes to progressive photoreceptor degeneration [19, 20]. Corneal involvement manifested as superficial opacities has also been described in *PEX*-related peroxisome biogenesis disorders [19]. Although cataracts were not specifically reported among patients homozygous for the c.1047_1049del variant, lens opacities are a recognized feature of Zellweger syndrome and related disorders [19–21].

Comparison of our patient with previously reported individuals carrying the *PEX12* c.1047_1049del variant further highlights both shared and distinctive clinical features **(**Table 2**).** Our patient demonstrates the core neurological manifestations consistently reported in this founder cohort, including hypotonia, gait disturbance, seizures, abnormal EEG findings, and sensorineural hearing loss. In contrast, some systemic and ectodermal features such as hepatomegaly and alopecia, described in other patients, were absent. Notably, although nystagmus has been reported in approximately three-quarters of affected individuals, it was not observed in our patient; instead, visual impairment associated with bilateral optic atrophy was present, expanding the phenotypic spectrum of this variant [21]. 

**Table 2:** Clinical features reported in patients carrying the *PEX12* c.1047_1049del (p.Gln349del) variant compared with the present case

Clinical feature	Previously reported patients (*n* = 20)*	Present case
Short stature	14 (≈ 70%)	Present
Underweight	10 (≈ 50%)	Present
Microcephaly	4 (≈ 20%)	Present
Hypotonia	19 (≈ 95%)	Present
Gait disturbance/incoordination	20 (100%)	Present
Hepatomegaly	5 (≈ 25%)	Not Present
Seizures	9 (≈ 45%)	Present
Abnormal EEG changes	7 (≈ 35%)	Present
Sensorineural hearing loss	7 (≈ 35%)	Present
Alopecia	17 (≈ 85%)	Not Present
Nystagmus	15 (≈ 75%)	Not Present
Visual impairment	9 (≈ 45%)	Present
Optic atrophy	Not reported	Present

*Clinical data were available for 19 of the 20 reported patients carrying the *PEX12* c.1047_1049del (p.Gln349del) variant

Data were compiled from previously published Egyptian cohorts and case series [9]. Percentages are approximate because clinical details were reported for only 19 patients, and one additional patient in the overall cohort carried a different *PEX12* variant. Absence of a feature indicates that it was not described in the original reports rather than definitively excluded

We hypothesize that the absence of reported optic atrophy in previous studies of patients carrying the *PEX12* c.1047_1049del variant may reflect a combination of disease heterogeneity, timing of manifestation, and ascertainment bias rather than a true absence of optic nerve involvement. Zellweger spectrum disorders are characterized by marked phenotypic variability and age-dependent progression, with clinical manifestations evolving across the neonatal, infantile, and childhood periods. It is therefore plausible that optic atrophy represents a later-onset feature that may not have been detectable at the time of earlier assessments. In addition, prior reports frequently attributed visual impairment in these patients to more readily identifiable anterior segment or retinal abnormalities, such as cataract or retinopathy, potentially limiting detailed evaluation of the optic nerve. The severe systemic condition, high morbidity and mortality associated with early-onset forms, and practical challenges in performing advanced ophthalmologic investigations (including neuro-ophthalmic imaging) may have further constrained comprehensive visual pathway assessment. Finally, incomplete longitudinal follow-up and underreporting of ophthalmologic findings in severely affected patients could have contributed to the lack of documented optic atrophy in earlier cohorts.

The main strength of this case report lies in the identification and detailed description of a rare, likely founder *PEX12* gene variant (c.1047_1049del) in an Egyptian patient, supported by comprehensive clinical, radiological, and genetic findings. This report also contributes novel insight by documenting optic atrophy for the first time in association with this specific variant.

However, the study has certain limitations, including the lack of confirmatory biochemical assays such as very-long-chain fatty acid (VLCFA) or plasmalogen quantification, and the absence of functional validation studies, mainly due to limited local testing facilities. In addition, formal assessment of retinal function, such as electroretinography (ERG) or Visual evoked potential (VEP), was not performed, which limits functional characterization of the reported optic involvement. Furthermore, as this is a single case report, it does not allow for broad causal inferences or genotype–phenotype correlations, which require larger multicentric studies to confirm.

To conclude, our patient has a homozygous *PEX12* variant (c.1047_1049del), and the clinical manifestations can be attributed to the essential role of peroxisomes in lipid metabolism and detoxification. So, the deficiency of functional *PEX12* disrupts peroxisome assembly, which impairs the breakdown of very-long-chain fatty acids (VLCFA) and the synthesis of plasmalogens as critical components of myelin and cell membranes. This results accumulation of toxic metabolites, cellular damage, and neurodegeneration. This metabolic disturbance explains the progressive hypotonia, developmental regression, and white matter abnormalities observed on MRI, as the central nervous system is particularly susceptible to disruptions in peroxisomal function. Similarly, the observed optic atrophy and sensorineural hearing loss are likely consequences of direct cytotoxic effects on retinal ganglion cells and auditory pathways caused by these accumulated metabolites [8, 22, 23].

This case expands the phenotypic spectrum of *PEX12*-related peroxisome biogenesis disorders, emphasizing the diagnostic value of whole-exome sequencing when biochemical results are inconclusive. Whole-exome sequencing has a high diagnostic yield for neurodevelopmental disorders and is particularly valuable when biochemical testing is nondiagnostic or clinical features are atypical. In populations with high consanguinity, awareness of this founder variant may facilitate earlier diagnosis, family counseling, and targeted carrier screening. Early recognition of ZSD should prompt multidisciplinary management aimed at preserving neurological function and preventing complications.

## Data Availability

All data generated or analysed during this study are included in this published article. The genetic variant identified in this study has been submitted to the ClinVar database (Submission ID: SUB16130941) and is currently under review. A public accession number will be provided once assigned by ClinVar. Additional clinical details are available from the corresponding author upon reasonable request.

## Abbreviations

ABR: Auditory Brainstem Response
ALT: Alanine Aminotransferase
AST: Aspartate Aminotransferase
BMC: BioMed Central
BW: Birth Weight
DWI: Diffusion-Weighted Imaging
EEG: Electroencephalography
EMG: Electromyography
ENT: Ear, Nose, and Throat
FLAIR: Fluid-Attenuated Inversion Recovery
GC–MS: Gas Chromatography–Mass Spectrometry
GTC: Generalized Tonic–Clonic
IRB: Institutional Review Board
MRI: Magnetic Resonance Imaging
NB: Narrow Band
NICU: Neonatal Intensive Care Unit
OMIM: Online Mendelian Inheritance in Man
PBD: Peroxisome Biogenesis Disorder
PEX: Peroxin (Peroxisome Biogenesis Factor)
SNHL: Sensorineural Hearing Loss
VLCFA: Very-Long-Chain Fatty Acids
WES: Whole-Exome Sequencing
ZSD: Zellweger Spectrum Disorder
